# The increasing use of T-cell stimulation for successful dengue vaccination

**DOI:** 10.1016/j.ebiom.2024.105120

**Published:** 2024-04-10

**Authors:** eBioMedicine

Dengue fever is a viral infection caused by the dengue virus, which contributes to tens of thousands of deaths annually. Endemic to tropical and subtropical regions, dengue virus is transmitted by *Aedes* mosquitoes that breed in warm stagnant waters. Upon biting a susceptible host, the virus is transferred to the bloodstream, leading, in most people, to fever and vomiting. At its worst, dengue virus can lead to internal bleeding and death. As an arbovirus (ie, an arthropod-borne virus), the number of dengue-virus infections in an area are often closely linked to mosquito breeding habits, leading to a cyclical pattern of widespread transmission spanning 3–4 years.

The expanding habitat of the *Aedes aegypti* mosquito due to climate change has made dengue a global health issue. A systematic review and meta-analysis published in *eBioMedicine* in April, 2023, identified that a 1°C increase in high temperatures corresponded to a 13% increase in dengue-virus infections. Furthermore, questions remain about even the most fundamental aspects of dengue-virus transmission, such as the possibility of so-called direct mechanical transfer from one individual to another via blood infected with dengue virus, which was shown in a mouse-model study published in *eBioMedicine* in July, 2023. Local interventions, such as netting over containers of standing water or improved drainage systems, can curb the growth of aquatic larval populations, although they do not constitute complete mosquito control.

On December 21, 2023, WHO reported the current situation of the global dengue-virus outbreak, citing that the reported annual number of cases had reached an unprecedented 5 million, increasing 10-fold from 500,000 in 2000. This report comes alongside concerns from the health ministry in Brazil, which predicted 5 million cases of dengue virus in Brazil alone during 2024. These statements, and the absence of any specific antiviral treatments against dengue fever, prompted the city of Dourados, Brazil to roll out the country's first-ever mass vaccination against dengue virus.

Development of safe and effective dengue virus vaccines, however, has been complicated by the presence of 4 serotypes (ie, dengue virus 1–4). Although they share almost 65% genome homology, infection with one serotype does not confer protection against the other three. In fact, subsequent heterotypic reinfection can trigger the process of antibody-dependent enhancement (ADE). In ADE, insufficiently protective concentrations of cross-reactive antibodies not only fail to neutralise heterotypic dengue virus, but can enhance its cellular invasion, increasing the likelihood of severe dengue symptoms.

The first-ever approved dengue virus vaccine for all four serotypes, the chimeric live attenuated CYD-TDV vaccine, encountered controversy upon being administered to children in the Philippines who had not previously been infected with dengue virus as it put them at risk of vaccine-induced ADE. Currently, WHO recommends the use of CYD-TDV only in people with a confirmed previous dengue-virus infection. However, this vaccine is only partly effective, suggested to be due to a poor cellular (ie, T-cell-mediated) immune response, as discussed in *Perspectives in Biology* in July, 2017.

An 18-month study of TAK-003, published in *The Lancet Infectious Diseases* in November, 2017, showed that the second generation of live-attenuated tetravalent vaccines were effective and safe for use in individuals without the requirement for stringent previous-infection testing. Since initial approval in Indonesia in August, 2022, TAK-003 has been approved in the EU, the UK, Argentina, Thailand, and Brazil. However, concerns persist that current safety data are insufficient and that the risk of ADE cannot be completely dismissed.

A review of studies published in April, 2014, emphasised the importance of T-cell responses in robust cellular immunity against dengue virus. These responses are typically directed against non-structural proteins, particularly NS3. Therefore, questions remain about whether TAK-003, which contains NS1–5 proteins based only on the dengue virus 2 serotype, elicits a serotype-independent cellular immunity. As such, current dengue-virus vaccinology increasingly focuses on the stimulation of dengue virus-specific T cells. Mouse models published in *eBioMedicine* in September, 2020, showed that a candidate DSV4 vaccine elicited a tetravalent antibody response without inducing ADE. A follow-up study published in *Frontiers in Immunology* in February, 2023, substantiated the notion that this DSV4 vaccine induced IFNγ secretion by both CD4^+^ and CD8^+^ T cells in mice, as well as stimulation of follicular CD4^+^ and type 1 helper T cells.

The possibility that dengue virus-specific T cells could be primed without eliciting a humoural response was published in *eBioMedicine* in December, 2023. In this double-blind, randomised, phase 1 study, gold nanoparticle-based peptides were administered to 20 participants. Six of the ten participants receiving the low-dose formulation were detected as having activation-induced marker-positive CD8^+^ T cells, whereas anti-dengue virus antibodies were only transiently detected in one individual across the entire study. Indications that the risk of ADE could be mitigated by this approach and the absence of serious vaccine-related adverse events further support the safety advantages of T cell-priming strategies. Whether this approach alone offers broad dengue virus protection remains to be shown in subsequent clinical trials. However, the possibility that T cell-boosting vaccines could be administered in combination with vaccines that enhance primarily humoural responses is of great importance to vaccination programmes, as discussed in *eBioMedicine* in February, 2024.

Both 2023 and 2024 are El Niño years, bringing warm and wet mosquito-favourable weather systems to regions with dengue virus. Together with the possible under-reporting of dengue during the COVID-19 pandemic, the coming years could be decisive in the defence against not only dengue fever and future ADE, but infections of the related yellow fever and Zika flaviviruses.

Development of the understanding and induction of robust T-cell immunity could be crucial for effective and long-lasting dengue virus vaccination. *eBioMedicine* encourages the submission of studies that investigate mechanisms of immunity and vaccination in dengue virus and associated flavivirus diseases.

